# A price tag on clinical empathy? Factors influencing its cost-effectiveness

**DOI:** 10.1177/0141076820945272

**Published:** 2020-09-15

**Authors:** J Howick, S Mittoo, L Abel, J Halpern, SW Mercer

**Affiliations:** 1 Faculty of Philosophy, 6396 University of Oxford, Oxford OX2 6GG, UK; 2 University Health Network, 7938 University of Toronto, Toronto, Ontario, M5G 2C4 Canada; 3 Nuffield Department of Primary Care Health Sciences, 6396 University of Oxford, Oxford OX2 6GG, UK; 4 School of Public Health, University of California at Berkeley, CA 94720-7360, USA; 5 Usher Institute, College of Medicine and Veterinary Medicine, University of Edinburgh, EH8 9AG UK

## Introduction

Good communication is a pillar of medical professionalism.^1^ As such, it is required independently of its economic cost. Yet, faced with scarce resources, it is useful to know the extent to which devoting resources to ensure or enhance high-quality communication is cost-effective. The benefits of enhancing one particular type of communication, namely empathic positive communication, have been studied in a number of randomised trials.^2^ These trials suggest that compared with ‘usual care’, empathic positive communication can reduce pain, improve patient satisfaction and increase quality of life. Observational studies suggest that empathic care has benefits ranging from reducing mortality^3^ and practitioner burnout^4^ to increasing safety.^5^ On the other hand, these very same studies reveal that additional time is required to undertake empathy training and to treat patients in the clinic; both of these can be costly. Unfortunately, with a few notable exceptions,^6^ empathy’s cost-effectiveness has not been rigorously evaluated. A definitive answer to whether practitioner empathy is cost-effective requires sufficiently powered trials or decision models that measure relevant outcomes, and this is starting to happen.^6^ In this overview, we consider the factors that might be weighed in future trials of empathy’s cost-effectiveness (see Table 1).

**Table 1.** Factors that potentially influence the cost-effectiveness of empathy.

	Factors likely to improve cost-effectiveness		Factors likely to worsen cost-effectiveness
*Direct benefits from randomised trials*	Reduced pain^15–17^	*Direct costs*	Additional time often required by practitioners^2^
	Improved quality of life^6,7^		
	Improved patient satisfaction^16,18–20^		
			
*Direct benefits from observational studies*	Reduced mortality by 50% among diabetic patients^3^		
	Improved wellbeing^6,21^		
	Reduced hospital duration of stay and readmission rates^22^		
*Indirect benefits*	Improved diagnosis^11^	*Indirect costs*	Training empathy^7,15^
	Improved medication adherence^13^		
	Reduced hospital admissions and psychiatric hospitalisation		
	Reduced patient stress.^23^ Lowered stress, in turn, may reduce pain, depression and anxiety and even lower the risk of heart disease^12^		
	Improved trust^24^ and subsequently medication adherence^13^		
*Benefits to practitioners*	Reduced practitioner burnout^4^		
	Reduced medico-legal risks^25^		

## Benefits of empathy (likely to favourably influence cost-effectiveness)

We are aware of two trials that measured the impact of enhanced empathic care on quality of life. The first measured participant-assessed quality of life, using the 34-question Irritable Bowel Syndrome Quality of Life score.^7^ In a sample of 262 patients, empathic care seemed to improve quality of life by a small, statistically significant amount (standardised mean difference 0.43 [95% confidence interval 0.13–0.73]). In another trial with 152 patients, a complex intervention which included empathy (as well as longer consultations, continuity of care and additional support to treat multimorbid patients) found that the quality of life was improved. In this study, quality of life measured over the 12-month period was higher in the intervention group (*p* = 0.002), and the intervention was highly cost-effective over the 12-month period. Modelling suggested that cost-effectiveness would continue.^6^

Pain (which enhanced empathy can reduce) strongly influences the quality of life measures commonly used to evaluate cost-effectiveness. Three trials (1067 patients) within a systematic review of empathic care found a small, non-statistically significant benefit (standardised mean difference −0.05 [95% confidence interval 0.32–0.22]).^2^

We found four trials (including 955 patients) reporting satisfaction as an outcome which suggested that this was improved by a small amount (standardised mean difference 0.26 [95% confidence interval 0.02–0.54]).^2^

Observational studies report (among other things) that enhanced empathy reduces mortality by 50% among diabetic patients,^3^ reduces symptom burden and improves wellbeing,^6^ increases patient enablement,^8^ increases patient safety,^5^ improves self-efficacy and adherence^9^ and reduces practitioner burnout.^4^

The mechanisms by which enhanced empathic care produces beneficial results are currently somewhat speculative. First, empathy can be helpful to make a correct diagnosis, as without it, patients may not share details of symptoms, especially embarrassing ones. They are also more likely to remain engaged.^10^ By contrast, doctors perceived to be unfriendly are less likely to get enough information from patients to make the right diagnoses or prescribe the right treatment. One study even showed that unempathic doctors could cause harm by scaring patients away from medical care when they need it.^11^ Next, an empathic doctor will help put a patient at ease and reduce their stress; lowered stress, in turn, may reduce pain, depression, anxiety and even lower the risk of heart disease.^12^ Being positive (which is part of empathic care) also activates the patient’s endogenous opioid system, further reducing pain. In addition, empathy seems to facilitate trust^10^ and subsequently medication adherence.^13^ Adherence, in turn, is linked to better clinical outcomes, with one study showing that up to 62% of patients were more likely to adhere to treatment based on the quality of physician communication.^14^ Anecdotally, those of us who are practitioners (SM, SWM and JoH) find that the benefits of enhanced empathic care could reduce hospital admissions and psychiatric hospitalisation. Patients seem more likely to visit empathic practitioners who can prevent more serious events like hospital admissions and also reduce fears about issues such as medication shortages and medical tests.

## Costs of empathy

The main way in which enhanced empathic care would increase healthcare costs is the potential additional time often required by practitioners to treat patients.^2^ However, the extent to which enhanced empathy requires more time is not clear. Some trials of enhanced empathy do not increase the consultation time by focusing on non-verbal communication.^19^

Another cost is the time and money spent training practitioners to enhance empathy. Some trials that trained practitioners to enhance their empathy required a team of professionals, video consultations and two days.^15^ However, trials that had much less (4 hours) training did not result in smaller patient benefits when compared with trials involving doctors that had more extensive training.^2^ While this training period amounts to a one-off cost whose marginal cost approaches zero, it nonetheless has the potential to be substantial.

## Other considerations for measuring cost-effectiveness

Better communication (which includes empathic communication) reduces medico-legal risks, which represents a further benefit.^25^ Some worry that a potential cost is increased practitioner burnout caused by the alleged increased emotional labour caused by empathic care. The evidence in this area, however, is mixed, with a growing consensus that *therapeutic* empathy reduces practitioner burnout and increases job satisfaction.^4^ Few things are likely to be more costly than losing physicians due to burnout and having to replace them. These long-term human resource outcomes are rarely included in cost-effectiveness analyses but may prove relevant in the evaluation of empathic interventions.

## Technology and the future of empathy’s cost-effectiveness

The costs in training and additional time spent in empathic care will be subject to change as technology and artificial intelligence becomes increasingly involved in healthcare.^26^ While it is unlikely that humanoid robots will be able to express empathy as well as real humans, the opportunity cost of training artificially intelligent robots (should they ever move out of the laboratory^27^) will be close to zero. They have to be trained in some way, so choosing to train them to be more empathic does not seem to represent an additional cost. Likewise, the growth of digital online learning (catalysed radically during the COVID-19 era) has the potential to reduce the marginal cost of empathy training, as online courses are highly scalable.

## Limitations to measuring cost-effectiveness

Measures of empathy’s cost-effectiveness will be as accurate as the underlying studies measuring its effects. The main problems with randomised trials of empathy are difficulty in blinding practitioners and subsequent contamination. Practitioners who are randomised to the control group of a trial of enhanced empathy know which group they are in, and their knowledge could affect the results. For example, the fact that they are aware that their empathy will be measured could lead them to be more empathic than they would usually be, thus decreasing the apparent benefits of empathy training. With that in mind, it is important to take the observational evidence into account, because contamination is not a confounder of observational studies.

To be sure, the results of observational studies have their own biases. A particularly salient potential problem with observational studies is selection bias. Patients who are healthier to begin with are more likely to be more sociable, less angry, easier to empathise with and more likely to do better over time than those who are less healthy to begin with. Still, the fact that all the observational studies reveal a large effect with a consistent direction is suggestive of a real effect.

The next main problem with trials of empathy is definition and measurement. One study identifyed 36 distinct ways to measure empathy within healthcare.^28^ In spite of this, there is an emerging consensus that within healthcare, *clinical* empathy has three components: understanding; demonstrating understanding; and therapeutic action.^29^ Relatedly, the two scales of clinical empathy that are most widely used – the Consultation and Relational Empathy (CARE) measure^30^ and the Jefferson Scale of Physician Empathy (JSE)^31^ – are compatible with the definition of clinical empathy.

## Discussion and recommendations

Beyond being a professional requirement, enhanced empathy has been proven in randomised clinical trials to serve two of the most important goals of medicine – decreasing pain and improving quality of life, by a small amount. Observational studies consistently show that empathy improves adherence to treatment, which is an important determinant of the cost-effectiveness of healthcare. Enhanced empathic care may also increase healthcare costs with resources required to encourage empathic care and additional time spent with patients. Our analysis found that there appear to be more potential benefits than costs.

Ultimately, whether empathy is cost-effective will depend on how robust all of its effects are, relative to all of its costs, over time. This can only be measured in comprehensive systematic cost-effectiveness analyses that take the factors listed here into account. Such analyses can weigh the factors listed in this paper. We recommend that a decision analytic model be used to evaluate potential cost-effectiveness using existing evidence, informing the design of future interventions and trials.

## Declarations

### Guarantor

JH.
